# Effect of the aqueous extract of the aerial parts of *Monsonia angustifolia* E. Mey. Ex A. Rich.*,* on the sexual behaviour of male Wistar rats

**DOI:** 10.1186/s12906-015-0880-4

**Published:** 2015-10-02

**Authors:** Gerda Fouche, Anthony J. Afolayan, Olubunmi A. Wintola, Tendani E. Khorombi, Jeremiah Senabe

**Affiliations:** Council for Scientific and Industrial Research (CSIR), Pretoria, South Africa; Medicinal Plants and Economic Development (MPED) Research centre, Department of Botany5, Alice, South Africa

**Keywords:** Aphrodisiac, *Monsonia angustifolia*, Ejaculation, Intromission, Sexual behaviour, Plant extracts

## Abstract

**Background:**

*Monsonia angustifolia* (Geraniaceae) is a medicinal plant traditionally used in South Africa to increase libido and to treat erectile dysfunction.

**Methods:**

*In-vivo* aphrodisiac activities of the crude extracts of the plant prepared in water at 3, 30 and 300 mg/kg body weight were evaluated for 7 days using sildenafil citrate (Viagra) and 1 % ethanol in distilled water as positive and negative controls respectively. Male rats were selected and monitored in each group for sexual behaviour by exposing them to sexually receptive females on days 1, 3 and 7 for 60 minutes each between 7:00 pm and 3:00 am. The following male sexual parameters were observed: Mount Frequency (MF), Intromission Frequency (IF), Mount Latency (ML), Intromission Latency (IL), Ejaculation Frequency (EF), Ejaculatory Latency (EL) and Post-Ejaculatory Interval (PEI).

**Results:**

The administration of the extract resulted in significant increase (*p* < 0.05) in mount frequency, intromission frequency, ejaculation frequency, ejaculation latency and serum hormone concentrations. The computed indices of sexual behaviour such as erection, quick flips, long flips and total penile reflexes were also increased. However, the mount latency, intromission latency and post ejaculation interval were significantly decreased throughout the experimental period. The administration of 300 mg/kg body weight of the aqueous extract produced the best effects in all the parameters.

**Conclusion:**

Generally, the extract of *M*onsonia *angustifolia* produced pro-sexual stimulatory effects in the male rats especially when administered at 300 mg/kg body weight. The results validate the use of the plant by the indigenous people to increase libido and treat premature ejaculation and erectile dysfunction in males.

## Background

Sexual behaviour is one of the fundamental principles of reproduction comprising mating, sexual behaviour, fertility, conception and reprocration. Several substances have been reported to stimulate or increase sexual desire and performance [1, 2]. These substances include Levodopa (dopamine), Amyl nitrite, Vitamin E and Sildenafil citrate (Viagra). Despite several reports on the use of these conventional substances, loss of sexual desire in male partners may be more common than arousal and orgasm disorder [3]. The sexual desire, erectile and fertility enhancing properties of plant extracts continue to increase in number. These include *Fadogia agrestis, Piper guineensis*, *Afromomum melegueta, Lepidium meyenii and Bulbine natalensis. B. Natalensis, Allium tuberosum, Terminalia catappa, Turnera diffusa , Cnestis ferruginea, Allium tuberosum, Curculigo orchioides* has been shown to have sexual and fertility enhancing properties [4]. The fertility reducing effects of medicinal plants such as *Ruta graveolens, Gossypol* have also been studied [4] *M. angustifolia* was claimed to have been used as an aphrodisiac in the folklore medicine of South Africa without much information on its effect on sexual behaviour and fertility in animal.

*M. angustifolia* E.Mey.ex. A. Rich. (Geraniaceae) is a medicinal plant used to increase male and female libido. It is widely distributed in the Eastern Cape, Free State, Gauteng, KwaZulu-Natal, Limpopo and Mpumalanga. The traditional remedy is taken as a decoction prepared from crushed dried leaves and stems of the plant. It is a plant listed among the National Assessment Red List of South Africa [5, 6]. The extract of the plant has demonstrated the potentiality of relaxing the rabbit corpus carvenosum smooth muscle [7]. Fractionation of the organic extract also led to the isolation of five compounds identified as lignans [7]. The four compounds showed minimum/non-cytotoxicity in the Chinese Hamster ovarian (CHO) toxicity assay at the effective concentrations.

The aqueous and organic extracts and compounds isolated from *M. angustifolia* have been evaluated in relevant *in-vitro* and *in-vivo* biological assays for erectile dysfunction and libido [7]. Many unpublished studies have reported the potential of the plant as a source of novel compounds for the development of new drugs [7]. CSIR Bioscience has established a protocol for the successful propagation of the plant for commercial purposes. However, there is a dearth of information in scientific literature, to the best of our knowledge, on the aphrodisiac effect of *M. angustifolia*. We believe that the commercialization of the products derived from the plant could be used to target the complementary medicine market thereby allowing a certain level of claim for efficacy in managing erectile dysfunction or the improvement of libido in males and females. It is with all this background that this study thus has the aim of investigating the aphrodisiac effect of *M. angustifolia* extract on the sexual behaviour of male Wistar rats.

## Methods

### Preparation of the aqueous extract of *M. angustifolia*

#### Collection of plant material of M. angustifolia

The aerial part of this scrub (1 kg) was collected near Chuenespoort in Polokwane area, Limpopo province. A plant specimen was sent to the South African Biodiversity Institute (SANBI) for identification. The plant was identified as *Monsonia angustifolia* E. Mey. ex A. Rich., voucher specimen number 582251.0

#### Aqueous extract of M. angustifolia

Plant material collected was dried in an oven at 60 °C until dry. The aerial parts were ground to a coarse powder using a hammer mill. 150.3 g dried, ground material was extracted using double distilled water (2 L) and boiled for one hour. The solution was filtered and the residual plant material was extracted again with 2 L of water. The water solution was left to cool to room temperature, filtered and both solutions combined and freeze-dried for 24 h to yield 10.13 g of a brownish fluffy powder.

### Experimental procedure

The experiment was carried out at the Animal House Unit of the Central Analytical Laboratory, University of Fort Hare. The animals were handled in accordance with the guidelines and principles of the University Ethical Committee. Animals were fed with Epol feed Chunks, South Africa, and tap water under hygienic conditions. Temperature was 23 ± 1 °C; photoperiod was 12 h natural light and 12 h dark while humidity was 45–50 %.

### Animal groupings and extract administration

Seventy-five (75) male Wistar rats (*Rattus norvegicus*) each weighing 270.00 ± 5.21 g and 75 females weighing 250.00 ± 4.35 g were bred and raised in the Animal House Unit. The male rats were completely randomized into 5 groups of 15 rats each. Sildenafil (trade name Viagra), is the first pharmacologically approved remedy for impotence, and its availability has brought millions of couples to erectile dysfunction treatment [3]. Hence, in this study, Viagra was used as the positive control drug and since the extracts were readily soluble in 1 % EtOH in water only, 1 % EtOH in water was used as the negative control. Oral administration of the extract (3, 30 and 300 mg/kg), Viagra (50 mg/kg) body weight and 6.7 mL⁄ kg of 1 % EtOH in water (1 ml) was done daily using metal gavaging cannula. Selection of the extract doses was based on previous studies [4, 8, 9] on rats using herbal extracts. Potential aphrodisiac activity and toxicity were considered for the selection of lowest (3 mg/kg) and highest 300 mg/kg) doses respectively.

The female rats were prepared and made receptive by the sequential administration of oestradiol benzoate (10 mg ⁄ 100 g body weight) and progesterone (0.5 mg/100 g body weight) through subcutaneous injections, 48 h and 4 h respectively, prior to pairing [10, 11].

### Observation of sexual behaviour

The male rats were trained by exposing them to sexually receptive females once daily for 4 consecutive days before the experiment. Each receptive female rat was introduced to a male rat for 30 min (adaptation period) in a metabolic cage (48.5 cm x 33.5 cm x 22.5 cm). The test was conducted after oral administration of the different doses of the extract and controls. The test was carried out and sexual behaviours were monitored in each group between 7:00 pm and 3:00 am under dim light 30 min after extract/drug administration on days 1, 3 and 7 [11].

### Measurement of sexual parameters

The following male sexual behaviour parameters were calculated after monitoring for 2 h: Mount Frequency (MF) which is the number of mounts from the time of introduction of the female until ejaculation. Intromission Frequency (IF) is the number of intromissions from the time of introduction of the female until ejaculation and Mount Latency (ML) is the time interval between the introduction of the female and the first mount by the male. Other parameters were Intromission Latency (IL), which was the time interval between the introduction of the female and the intromission by the male, Ejaculation Frequency (EF), is the number of ejaculations from the time of introduction of the female rats to the male within a given time interval (30 min), Ejaculatory Latency (EL) which is the time interval between the first intromission and ejaculation and Post-Ejaculatory Interval (PEI) which is the time interval between an ejaculation and the next intromission. Sexual behaviour parameters were calculated as described by [8, 12] as follows; % index of libido = (number mated ⁄ number paired) x 100; % mounted = (number mounted ⁄ number paired) x 100; % intromitted = (number intromitted ⁄ number paired) x 100; % ejaculated = (number ejaculated ⁄ number paired) x 100; % copulatory efficiency = (number of intromissions ⁄number of mounts) x 100; Intercopulatory efficiency (s) = average time between intromissions; Intromission ratio or copulatory efficiency = (number of intromissions⁄ number of mounts + number of intromissions).

### Test for penile reflexes

The test for penile reflexes was carried out as described by Amin et al. [11]. Half an hour (30 min) after administering the doses on the 8th day, the animals were placed on their back in a glass cylinder with partial restraint. The pre-putial sheath was pushed behind the glands and held in this manner for a period of 15 min. The frequency of the following components of penile reflexes was recorded ⁄calculated: Erections (E), Quick Flips (QF), Long Flips (LF) and Total penile reflexes (TPR) were determined as TPR = E + QF + LF.

### Hematological parameters

At the end of the experiment, the rats were humanly sacrificed under iso-flourine anaesthesia and the blood was collected and sent to National Health Laboratory through Victoria Hospital and Pathcare Diagnostic Veterinary Laboratory for analysis of the various haematological parameters including serum testosterone and luteinizing hormonal assays.

### Ethical approval

Ethical approval for the study was granted by the University of Fort Hare Ethical Committee on the 27/11/2012 with certificate number AFO022.

### Statistical analysis

A completely randomised design was used in this study. Data were expressed as means of three replicates ± SD. Where necessary, data are represented as mean ± standard error of mean and were analyzed using a one-way analysis of variance (ANOVA) and complemented with Student’s t-test. All the statistical analyses were done using Minitab Student release version 12, Windows 95. Significant levels were tested at *P* < 0.05.

## Results

The male rats in all the groups advanced towards the females on introduction into the cages with precopulatory behaviours such as chasing and or anogennital sniffing which eventually resulted to mounting, intromission and ejaculation. The rats also did not show any indication of tiredness which was an apparent manifestation that the extract did not produce sedative effect throughout the observatory period.

**Fig. 1:**
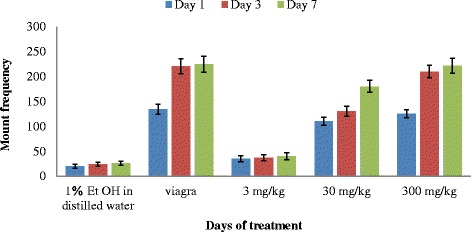
Effect of aerial aqueous extract of *M. angustifolia* on mount frequency in Wistar rats

The highest mount frequency (MF) was recorded in the rats administered with 300 mg/kg body weight and Viagra, followed by those administered with 30 mg/kg and least in those administered with 3 mg/kg (Fig. 1). The MF of rats administered with 3 and 30 mg/kg increases with days of administration like the positive control (viagra). The highest extract dose (300 mg/kg) significantly increased (*P* <0.05) the mount frequency from the 1st day to day 3 and this then reduced slightly on day 7. The two way Anova showed that duration of treatment on rats with extracts did not have any significant effect (*P* = 0.19) on mount frequency.

**Fig. 2:**
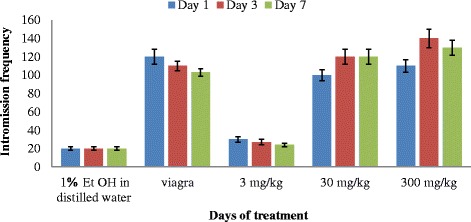
Effect of aerial aqueous extract of *M. angustifolia* on intromission frequency in Wistar rats

The highest intromission frequency (IF) was observed in rats that were administered with 300 mg/kg bw of the extract followed by 30 mg/kg and 3 mg/kg respectively (Fig. 2). The sample with the highest extract concentration induced the highest frequency of intromission and the intromission frequencies decreased with corresponding decrease of extract concentration. The frequencies of intromission in the rats administered with 30 and 300 mg/kg of the extract was not significantly different (*P* < 0.05) when compared with Viagra on the 1st day of administration. The two way Anova showed that duration of treatment on rats with extracts did not have any significant effect (*P* = 0.23) on intromission frequency.

**Fig. 3:**
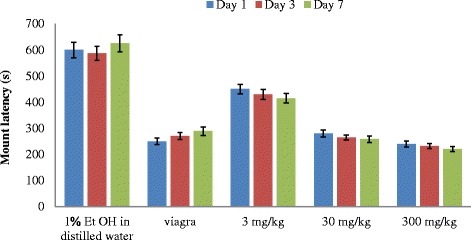
Effect of aerial aqueous extract of *M. angustifolia* on mount latency in Wistar rats

Mount latency (ML) shows the time interval from the introduction of the female up to the first mount (Fig. 3). The mean mount latency was highest in the rats administered with 3 mg/kg body weight on all the days of administrations followed by those administered with 30 mg/kg body weight of the extract throughout the period of experiment. While the mount latency of the rats administered with 3 mg/kg reduces from day 1–7, the ML of the rats administered with 30 mg/kg bw increases on day 3 and later reduced on day 7. The rats administered with the highest concentration (300 mg/kg) bw of the extract however, showed the least mount latency among the three concentrations with an increase in ML on day 3 and a decrease on day 7. The mount latency induced by the highest concentration of the aqueous extract was not significantly different (*P* < 0.05) from that of the positive control drug (Viagra) whose ML increases as the days of administration increases. The extract significantly decreased the latency of mount especially when compared with the negative control (1 % EtOH in distilled water) (Fig. 3). The two way Anova showed that duration of treatment on rats with extracts did not have any significant effect (*P* = 0.17) on mount latency.

**Fig. 4:**
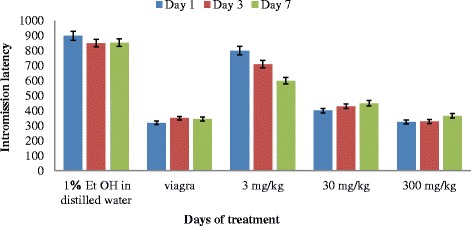
Effect of aerial aqueous extract of *M. angustifolia* on intromission latency in Wistar rats

There is a general decrease in the mean IL with increasing concentration of extract (Fig. 4). The effect of Viagra on intromission latency of the male rats was not significantly different (*P* < 0.05) from the extract at 30 and 300 mg/kg respectively on all the days of intervention. The IL of the rats administered with the extracts at 30 and 300 mg/kg increased with days of administration from the 1st to the 3rd day, while the IL of the rats administered with viagra increased only on days 1 to 3, this then stabilises till day 7. The rats administered with 3 mg/kg dose of the extract had reduced intromission latency up to the last day of administration. The two way Anova showed that duration of treatment on rats with extracts did not have any significant effect (*P* = 0.83) on intromission latency.

**Fig. 5:**
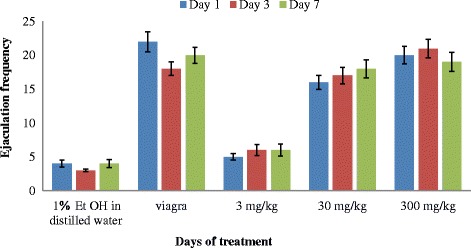
Effect of aerial aqueous extract of *M. angustifolia* on ejaculation frequency in Wistar rats

The highest mean ejaculation frequency (EF) was observed in the male rats administered with 300 mg/kg and was not significantly different (*P* < 0.05) from that of the viagra, followed by those administered with 30 mg/kg and 3 mg/kg (Fig. 5). The ejaculation frequency increased with concentration. Apart from the reduced ejaculation frequency produced by the rats administered with 30 mg/kg bw of the extract on the first and third days of administration, the value at the end of the experimental period was significantly increased on day 7 (Fig. 5). The ejaculation frequency produced by the rats administered with the 300 mg/kg weight of the extract increased on day 3, and reduces slightly on day 7. There is also an increase in the ejaculation frequency of the rats administered with the 3 mg/kg on all the days of administration. However, there was no significant difference in the rats administered with the 30 and 300 mg/kg of the extracts and Viagra. The two way Anova showed that duration of treatment on rats with extracts did not have any significant effect (*P* = 0.44) on ejaculation frequency.

**Fig. 6:**
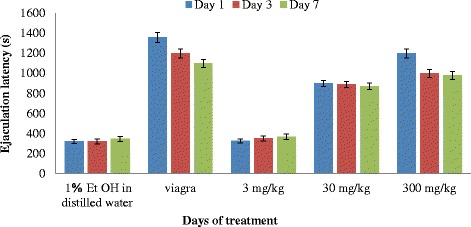
Effect of aerial aqueous extract of *M. angustifolia* on ejaculation latency in Wistar rats

The mean Ejaculation latency (EL) of the rats administered with the positive drug was higher than the rats administered with 300 mg/kg of the extract (Fig. 6), this was however not significantly different (*P* < 0.05) from the highest dose of the extract. The EL of the rats administered with 3 and 30 mg/kg body weight of the extract was significantly lower (*P* < 0.05) from the rats administered with 300 mg/kg. The control (1 % EtOH in distilled water) showed the least ejaculation latency. The two way Anova showed that duration of treatment on rats with extracts did not have any significant effect (*P* = 0.51) on ejaculation latency.

**Fig. 7:**
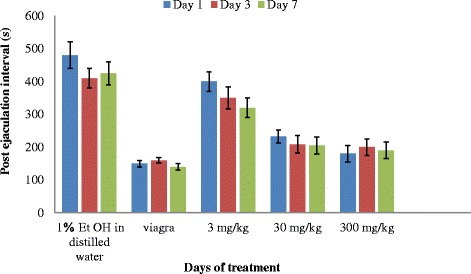
Effect of aerial aqueous extract of *M. angustifolia* on post ejaculation interval in Wistar rats

There was a general decrease in PEI observed in the rats treated with the highest extract concentrations (Fig. 7). The positive drug (Viagra) however followed the trends observed in the rats administered with 30 and 300 mg/kg bw with no significant difference in the days of administrations. The rats administered with the positive drug showed significant difference (*P* < 0.05) to the rats administered with the 3 mg/kg bw of the extract. The rats administered with viagra and the highest concentration of the extract had an increase in PEI from days 1–3 and this later decreases slightly on day 7, while those rats administered with 30 mg/kg bw showed a decrease PEI from day 1–3 and this later stabilises, those administered with 3 mg.kg bw recorded a decrease in PEI on all the days of administration. The two way Anova showed that duration of treatment on rats with extracts did not have any significant effect (*P* = 0.38) on post ejaculation interval.

The index of libido was the same (100 %) in all the rats administered with the extracts and Viagra and (80 %) in the rats administered with 1 % EtOH in water (Table 1). Similarly, the index of mount was the same in rats administered with the aqueous extracts and Viagra. The least mount index was recorded in rats administered with 1 % EtOH in water. Also the index of intromission was the same in the rats administered with 300 and 30 mg/kg extract and Viagra. The intromitted ratio was highest in rats administered with 300 mg/kg aqueous extract, followed by Viagra.

**Table 1:** Computed male rat sexual behaviour parameter. Data in means of 9 replicates ± SD

Parameters/ doses	% index of libido	% mounted	% intromitted	intromitted ratio	% ejaculated	copulatory efficiency	intercopulatory interval (sec)
Control (1 % EtOH in water)	80	80	80	0.33	70	66.8	172.5 ± 0.50^a^
Viagra (50 mg/kg bw)	100	100	100	0.42	100	82.8	75.0 ± 0.51^b^
3 mg/kg bw	100	100	90	0.39	90	75.5	135.1 ± 0.25^c^
30 mg/kg bw	100	100	100	0.40	100	78.0	89.7 ± 0.26^b^
300 mg/kg bw	100	100	100	0.41	100	80.9	81.0 ± 0.10^b^

^a-c^ Test value carrying superscript different from the control are significantly different (*P* < 0.05)

The ejaculation percentage was the same in rats administered with 300 and 30 mg/kg aqueous extracts and Viagra. The least percentage ejaculation (70 %) was observed in the rats administered with 1 % EtOH in water. The copulatory efficiency was highest in rats administered with Viagra and 300 mg/kg aqueous extract. The intercopulatory interval was highest in the rats administered with the 3 mg/kg of the extract and the negative control. The rats administered with the 30 and 300 mg/kg bw of the extract have the lowest copulatory interval, this was however, not significantly different (*P* < 0.05) from rats that were administered with Viagra.

Erections and quick flip in the rats administered with the extracts and Viagra were not statistically different (*P* < 0.05) from one another, while the long flips were higher in only the rats administered with the 300 and 30 mg/kg and lower in 3 mg/kg bw of the extract. The 30 and 300 mg/kg body weight extract significantly enhanced the total penile reflexes and its components, whereas the value produces by the 3 mg/kg body weight of the extract was significant different from Viagra and not significantly different from the negative control.

**Fig. 8:**
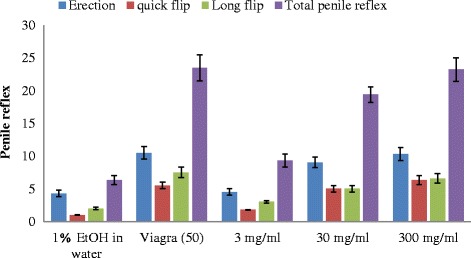
Effect of aerial aqueous extract of *M. angustifolia* on penile reflex in Wistar rats

**Fig. 9:**
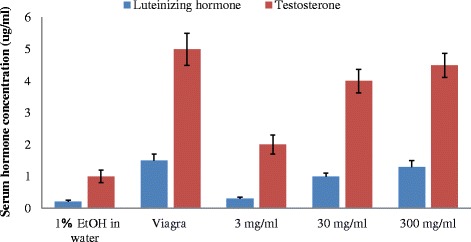
Effect of aerial aqueous extract of *M. angustifolia* on serum hormones concentration in Wistar rats

The total penile reflex (TPR) was generally higher in male rats administered with 300 mg/kg bw of the extract and Viagra for all the penile parameters investigated (Fig. 8). This was followed by the rats administered with 30 mg/kg and least in those administered with 3 mg/kg bw of the extract. The level of serum testosterone and luteinizing hormone concentrations in rats administered with the 30 and 300 mg/kg bw of the extract were significantly higher (*P* < 0.05) than those of the rats administered with 3 mg/kg bw of the extract (Fig. 9). The level of these hormones in male rats administered with Viagra was not significantly different (*P* < 0.05) from those rats administered with the extracts at 30 and 300 mg/kg, but to those administered with 3 mg/kg bw of the extract. Apart from the positive drug, the level of the serum testosterone and luteinizing hormone concentrations increased with increasing concentration of the extract.

The computed sexual behaviour parameters of the rats are shown in Table 1. The rats administered with the extract significantly increased all the sexual behaviour parameters except rats administered with 3 mg/kg body weight of the extract showing reduced intromission and ejaculation percentages.

Result of the two way ANOVA shows that the second factor ''time'' did not show any significant effect on any of the sexual parameters investigated.

## Discussion

Drug development from plants has formed the basis of *in vivo* research in recent years. This is because of their importance and contributions to human health and well-being most especially in reproductive health. Plants have been used globally across varied cultures as a safe natural source of medicines to contest the recurrent deterioration in sexual behaviour [4, 13]. Several plant products increased male and female fertility, hence the need to screen more botanicals resources of natural origin to assist in remediating the increase in sexual dysfunction globally.

Mount frequency (MF) and intromission frequency (IF) are useful indices of vigour, libido and potency. While the number of mount (MF) reflects sexual motivation, increase in the number of intromission (IF) shows the efficiency of erection, penile orientation and the ease by which ejaculatory reflexes are activated [9, 14]. The increase in mount frequency (MF) and intromission frequency (IF) following the administration of the aqueous extract of *M. angustifolia*, at 30 and 300 mg/kg body weight suggests improved sexual vigour/libido. Similar finding was also recorded by Tajuddin et al. [15], while working on ethanolic extracts of *Myristica fragrans* and *Syzygium aromaticum* in male rats.

The increase in IF of the male rats induced by the extract in this study suggests that the penile erection was activated. Therefore, extract of *M. angustifolia* may increase potency by allowing or sustaining erection. Plant activities on penile erection have been attributed to the various phytochemicals, like the alkaloids and saponins, in plants which have erogenic properties in vasodilation of the blood vessels and consequent erection [14, 16]. A report by Kim et al. [16] showed the induced relaxation of the corpus cavernosum muscle by the saponin content of *Panax ginseng* acting as nitric oxide donor through the L-arginine/nitric oxide pathway. Disparity in the values of MF and IF in this study suggests that it was not every mount by the male rats that resulted in intromission.

Mount latency (ML) and intromission latency (IL) are indicators of sexual motivation [4]. Both parameters are inversely proportional to sexual motivation. Therefore, the decrease in the mount and intromission latencies observed at the doses of 30 and 300 mg/kg body weight in this study might imply stimulation of sexual motivation and arousal. It may also be an indication of enhanced sexual appetitive behaviour in the male rats which further supports the sexual improvement effect of the extract. The higher mount latency observed in the rats administered with the lower dose of the extract is an indication in the hesitation time of the male rats towards the receptive females [4]. This agreed with the findings of Yakubu et al. [17], on *Massularia acuminata* root in male Wistar rats at the concentrations of 25, 50 and 100 mg/kg body weight.

The increase in ejaculation frequency by the extract of *M. angustifolia* at 30 and 300 mg/kg body weight is an indication of enhanced aphrodisiac effect of the plant. The dose dependent increase in the frequencies of mount and intromission in the rats administered with the extract were indications that the extract has the potential to control erectile dysfunction and arousal disorders in males.

Prolongation of the ejaculatory latency by itself suggests an aphrodisiac action. All the treated rats mounted and intromitted without any inhibition of mount-and-intromission frequencies or copulatory efficiency or intercopulatory interval. This suggests that libido, sexual vigour and sexual performance were unimpaired during the aphrodisiac action. The significant increase in ejaculation latency (EL) suggests that the extracts and standard drug prolonged the duration of coitus, which is an indicator of increase in sexual motivation [18]. The prolonged ejaculation latency of the extract of *M. angustifolia* at 30 and 300 mg/kg bw is an indication that copulatory performance in the animals was enhanced. Similar findings have been reported on the extracts of *Allium tuberosum* [19] and *Moringa oleifera* [12].

The post ejaculatory interval (PEI) is considered an index of potency, libido and the rate of recovery from exhaustion after first series of mating [15]. It is an important parameter for evaluating the effect of administered extracts on erectile function. [20, 21]. The significant increase in ejaculation latency (EL) suggested that the extracts and standard drug prolonged the duration of coitus, which is an indicator of increase in sexual motivation [22]. The decreased PEI observed in the *M. angustifolia* extract treated groups indicates potency and libido enhancement or less exhaustion in the first series of mating or both. Decrement of PEI is a reflection of the improvement of erectile function and the ability to perform better copulation.

In humans, it has been shown that penile sensitivity is altered in men suffering from premature ejaculation compared with controls [23]. Similar findings have been reported by Pattij et al. [24] on the study of the differences in the ejaculatory behaviours of individual rats in ejaculation disorders using medicinal plant for the treatment of premature ejaculation in traditional medicine. The similarity in the values of the sexual parameters in the extract of *M. angustifolia* and sildenafil citrate dosed groups suggests similar aphrodisiac activity.

Enhancement of libido might have arisen from the increase in concentration of anterior pituitary hormones and serum testosterone. These hormones are believed to stimulates dopamine receptor synthesis as a key neurotransmitter in the control of locomotor activity essential for the display of copulatory and sexual behaviour [14, 25, 26]. Testosterone enhances sexual desire, motivation and sexual performance. Mating on the other hand is associated with increasing the circulation of testosterone during the acquisition of sexual experience [27]. The rats in this study mated over a two-day period within a week and would thus have experienced repeated increases in levels of testosterone [28].

The enhanced copulatory performance of male rats by the extract of *M. angustifolia* may be attributed to the increase in serum testosterone concentration as revealed in this study. In a complex mechanism that regulates copulatory behaviour, testosterone is considered to have contributed to the improvement in sexual function, libido and penile erection [4]. In a similar trend, the presence of significant effect of the extract on the luteinizing hormones (LH) at the highest doses investigated may lead to the stimulation of the hypothalamic–pituitary–gonadal axis. Increases in LH, FSH, and testosterone levels also indicate an effect of *M. angustifolia* extract on gonadotropin release hormone (GnRH). In the testes, LH binds to receptors on Leydig cells, stimulating synthesis and secretion of testosterone [29]. A critical level of blood testosterone is required for the maintenance of normal sexual desire and non-erotic penile erections in most men while the combination of FSH and testosterone is qualitatively and quantitatively responsible for fully normal spermatogenesis probably mediated by the hypothalamic GnRH system [30].

The significant increase in the computed parameters observed with the rats administered with 30 and 300 mg/kg body weight of the extract is an indication of significant and sustained increase in the sexual activity and aphrodisiac property inherent in the plant extract. The median lethal dose (LD_50_) of the leaf extract of *M. angustifolia* reveals that the plant is safe up to 300 mg/kg as no death was recorded at this dose [31].

The effectiveness of the plants used as aphrodisiac is believed to be through various mechanisms such as vasodilation, elevation of androgens, gonadotropin and generation of nitric oxide [14, 17]. In this study, treatment of the male rats with the extract of *M. angustifolia* enhanced sexual behaviour of the male rats administered with 30 and 300 mg/kg body weight of the extract, producing better results than the 3 mg/kg body weight. There were no clinical signs and defects of toxicity, stress or changes in behaviour and appearance throughout the study. The food and water intake of all treated rats was similar to those of the control.

## Conclusions

Generally, the extract of *M. angustifolia* produced pro-sexual stimulatory effects in the male rats especially when administered at 300 mg/kg body weight. The results validate the use of the plant by the indigenous people for the treatment of disorders of desire/libido, premature ejaculation and erectile dysfunction in males.

## Abbreviations

MF: Mount Frequency
IF: Intromission Frequency
ML: Mount Latency
IL: Intromission Latency
EF: Ejaculation Frequency
EL: Ejaculatory Latency
PEI: Post-Ejaculatory Interval

## References

[1] Singh G, Mukherjee T (1998). Herbal aphrodisiacs: a review. Indian Drugs.

[2] Chauhan NS, Sharma V, Dixit VK, Thakur M. A Review on Plants Used for Improvement of Sexual Performance and Virility. Biomed Res Int. 2014;1–19. 10.1155/2014/868062 PMC4151601 25215296

[3] Potts A, Gavey N, Grace VM, Vares T (2003). The downside of Viagra: women’s experiences and concerns. Soc Health & Illness.

[4] Yakubu MT, Afolayan AJ (2009). Effect of aqueous extract of *Bulbine natalensis* (Baker) stem on the sexual behaviour of male rats. Int J Androl.

[5] Foden W, Potter L. Monsonia angustifolia E. Mey. Ex A. Rich. National Assessment: Red List of South Africa Plant Version 1 (http://redlist.sanbi.org/species.php?species=1979-2). Accessed on 2015/09/15, 2012.

[6] Raimondo D, von Staden L, Foden W, Victor JE, Helme NA, Turner RC, et al. *Red List of South African Plants. Strelitzia* 25. Pretoria: South African National Biodiversity Institute; 2009. eds.

[7] Khorombi TE. A chemical and pharmacological investigation of three South African plants. M.Sc dissertation, University of KwaZulu Natal South Africa, Pietermaritzburg, School of Chemistry; 2006

[8] Yakubu MT. Aphrodisiac potentials and toxicological evaluation of aqueous extract of Fadogia agrestis (Schweinf. Ex Hiern) stem in male rats. Ph.D. Thesis, University of Ilorin, Ilorin, Nigeria; 2006

[9] Yakubu MT, Akanji MA. Effect of Aqueous Extract of *Massularia acuminata* Stem on Sexual Behaviour of Male Wistar Rats. Evidence-Based Complement Alternate Med 2010*,* Article ID 738103, 10 pages doi:10.1155/2011/738103Article ID 738103, 10 pages doi:10.1155/201. 10.1155/2011/738103 PMC3022175 21253466

[10] Szechtman Z, Moshe H, Rabi S (1981). Sexual behaviour pain sensitivity and stimulates endogenous opoid in male rats. Eur J Pharmacol.

[11] Amin KMY, Khan MN, Rahman SZ, Khan NA (1996). Sexual function improving effect of *Mucuna pruriens* in sexually normal male rats. Fitoterapia.

[12] Zade VS, Dabhadkar DK, Thakare VG, Pare SR (2013). Effect of aqueous extract of Moringa oleifera seed on sexual activity of male albino rats. Int J Bio Forum.

[13] Adimoelja A (2000). Phytochemicals and the breakthrough of traditional herbs in the management of sexual dysfunction. Int J Androl.

[14] Ratnasooriya WD, Dharmasiri MG (2000). Effects of Terminalia catappa seeds on sexual behaviour and fertility of male rats. Asian J Androl.

[15] Tajuddin A, Ahmad S, Latif A, Qasmi IA. Effect of 50 % ethanolic extract of *Syzygium aromaticum* (L.) Merr. & Perry. (clove) on sexual behaviour of normal male rats. BMC Complement Altern Med. 2003;3:6. 10.1186/1472-6882-4-17 PMC534794 15530165

[16] Kim HJ, Woo DS, Lee G, Kim JJ (1998). The relaxation effects of ginseng saponin in rabbit corporal smooth muscle, is it a nitric oxide donor. British J Urol.

[17] Yakubu MT, Awotunde OS, Ajiboye TO, Oladiji AT, Akanji MA (2010). Pro-sexual effects of aqueous extracts of *Massularia acuminata* root in male Wistar rats. Andrologia.

[18] Wattanathorn J, Pangphukiew P, Muchimapura S, Sripanidkulchai K, Sripanid kulchai B (2012). Aphrodisiac activity of *Kaempferia parviflora*. Ame J Agric Bio Sci.

[19] Guohua H, Yanhua I, Rengang M, Dongzhi W, Zhengzhi M, Hua Z (2009). Aphrodisiac properties of *Allium tuberosum* seeds extract. J Ethnopharmacol.

[20] Thakur M, Chauhan NS, Bhargava S, Dixit VK (2009). A comparative study on aphrodisiac activity of some Ayurvedic herbs in male albino rats. Arc Sexual Behaviour.

[21] Sharma V, Thakur M, Chauhan NS, Dixit VK (2010). Effects of petroleum ether extract of *Anacyclus pyrethrum* DC on sexual behaviour in male rats. J Chinese Integrate Med.

[22] Wu D, Gore AC (2010). Changes in androgen receptor, estrogen receptor alpha, and sexual behaviour with aging and testosterone in male rats. Hormones and Behaviour.

[23] Rowland DL (1998). Penile sensitivity in men: a composite of recent findings. Urology.

[24] Pattij T, De Jong TR, Uitterdijk A, Waldinger WD, Veening JG, Cools AR, et al. Individual differences in male rat ejaculatory behaviour: searching for models to study ejaculation disorders. Eur J Neurosci. 2005;22:724–34. 10.1111/j.1460-9568.2005.04252.x 16101754

[25] Giuliano F, Allard J (2001). Dopamine and male sexual function. Eur Urol.

[26] Bahmanpour S, Talaei T, Vojdani Z, Panjehshahin MR, Poostpasand A, Zareei S, et al. Effect of phoenix dactylifera pollen on sperm parameters and reproductive system of adult male rats. Int J Molecular Sci. 2006;31:208–12.

[27] Swaney WT, Dubose BN, Curley JP, Champagne FA (2012). Sexual experience affects reproductive behaviour and preoptic androgen receptors in male mice. Hormones and Behaviour.

[28] Loomis T (1993). Essentials of toxicology.

[29] Chauhan NS, Sharma V, Thakur M, Dixit VK (2013). *Pueraria tuberosa* DC Extract improves androgenesis and sexual behavior via FSH LH Cascade. Scientific World Journal.

[30] Chauhan NS, Saraf DK, Dixit VK (2010). Effect of vajikaran rasayana herbs on pituitary–gonadal axis. European Journal of Integrative Medicine.

[31] Dewsbury DA, Davis HN (1970). Effect of reserprine on the copulatory behaviour of male rats. Physiol Behaviour.

